# Development and Validation of a Clinical-Image Model for Quantitatively Distinguishing Uncertain Lipid-Poor Adrenal Adenomas From Nonadenomas

**DOI:** 10.3389/fonc.2022.902991

**Published:** 2022-07-13

**Authors:** Wenting Pan, Huangqi Zhang, Shengze Jin, Xin Li, Jiawen Yang, Binhao Zhang, Xue Dong, Ling Ma, Wenbin Ji

**Affiliations:** ^1^ Department of Radiology, Taizhou Hospital of Zhejiang Province Affiliated to Wenzhou Medical University, Taizhou, China; ^2^ Department of Radiology, Taizhou Hospital of Zhejiang Province, Shaoxing University, Taizhou, China; ^3^ Department of Radiology, Taizhou Hospital, Zhejiang University, Taizhou, China; ^4^ He Kang Corporate Management (Shanghai) Co.Ltd, Shanghai, China

**Keywords:** adrenal adenoma, computed tomography, model, clinic, distinguish

## Abstract

**Background:**

There remains a demand for a practical method of identifying lipid-poor adrenal lesions.

**Purpose:**

To explore the predictive value of computed tomography (CT) features combined with demographic characteristics for lipid-poor adrenal adenomas and nonadenomas.

**Materials and Methods:**

We retrospectively recruited patients with lipid-poor adrenal lesions between January 2015 and August 2021 from two independent institutions as follows: Institution 1 for the training set and the internal validation set and Institution 2 for the external validation set. Two radiologists reviewed CT images for the three sets. We performed a least absolute shrinkage and selection operator (LASSO) algorithm to select variables; subsequently, multivariate analysis was used to develop a generalized linear model. The probability threshold of the model was set to 0.5 in the external validation set. We calculated the sensitivity, specificity, accuracy, and area under the receiver operating characteristic curve (AUC) for the model and radiologists. The model was validated and tested in the internal validation and external validation sets; moreover, the accuracy between the model and both radiologists were compared using the McNemar test in the external validation set.

**Results:**

In total, 253 patients (median age, 55 years [interquartile range, 47–64 years]; 135 men) with 121 lipid-poor adrenal adenomas and 132 nonadenomas were included in Institution 1, whereas another 55 patients were included in Institution 2. The multivariable analysis showed that age, male, lesion size, necrosis, unenhanced attenuation, and portal venous phase attenuation were independently associated with adrenal adenomas. The clinical-image model showed AUCs of 0.96 (95% confidence interval [CI]: 0.91, 0.98), 0.93 (95% CI: 0.84, 0.97), and 0.86 (95% CI: 0.74, 0.94) in the training set, internal validation set, and external validation set, respectively. In the external validation set, the model showed a significantly and non-significantly higher accuracy than reader 1 (84% vs. 65%, P = 0.031) and reader 2 (84% vs. 69%, P = 0.057), respectively.

**Conclusions:**

Our clinical-image model displayed good utility in differentiating lipid-poor adrenal adenomas. Further, it showed better diagnostic ability than experienced radiologists in the external validation set.

## Introduction

Over the last decades, there has been an epidemic increase in the detection of adrenal incidentalomas (1). Adrenal adenomas account for most adrenal lesions and do not require further treatment or only need regular follow-up (2). Adrenal adenomas that contain large amounts of fat could be reliably diagnosed through conventional imaging methods (3). However, 30% of adenomas having an attenuation value of >10 HU (i.e., lipid-poor adenomas) cannot be correctly differentiated from nonadenomas (1). For adrenal lesions suspected to be metastatic tumors or pheochromocytoma, further clinical examination and intervention are needed to avoid adverse events, such as life-threatening hypertension crises during operation. Therefore, it is important to distinguish adrenal lipid-poor adenoma from nonadenoma.

Chemical shift magnetic resonance imaging and energy spectrum computed tomography (CT) are slightly more sensitive for detection (4–7). However, their general use is limited by the high price and relatively limited accessibility. Thus, lipid-poor adrenal lesions usually need a dedicated adrenal washout CT protocol for further characterization (8–11). Nevertheless, the delayed phase and additional radiation exposure may limit the utility of the washout CT protocol (12). However, the relative percentage wash-in ratio of adrenal lesions from the unenhanced to the portal venous phase can remedy the above defects (12, 13). To our knowledge, only a few studies including a large number of lipid-rich adenomas have simultaneously assessed unenhanced attenuation and contrast wash-in features on CT (12, 14). There is currently no combined model established on easily available demographic information and CT characteristics for distinguishing lipid-poor adenomas and nonadenomas. Therefore, we aimed to develop a practical clinical-image model for identifying lipid-poor adrenal lesions.

## Materials and Methods

Our study was approved by the Institutional Review Committee, and the requirement of written informed consent was waived. We followed the TRIPOD Statement (15) and completed the checklist (**Supplementary Table S1**).

### The Training Set and the Internal Validation Set

We conducted a retrospective study on patients with adrenal lesions who were continuously treated in Institution 1 from January 2015 to August 2021. The inclusion criteria were as follows: adult patients with adrenal lesions who underwent adrenal or abdominal unenhanced and contrast-enhanced CT scans.

**Figure 1:**
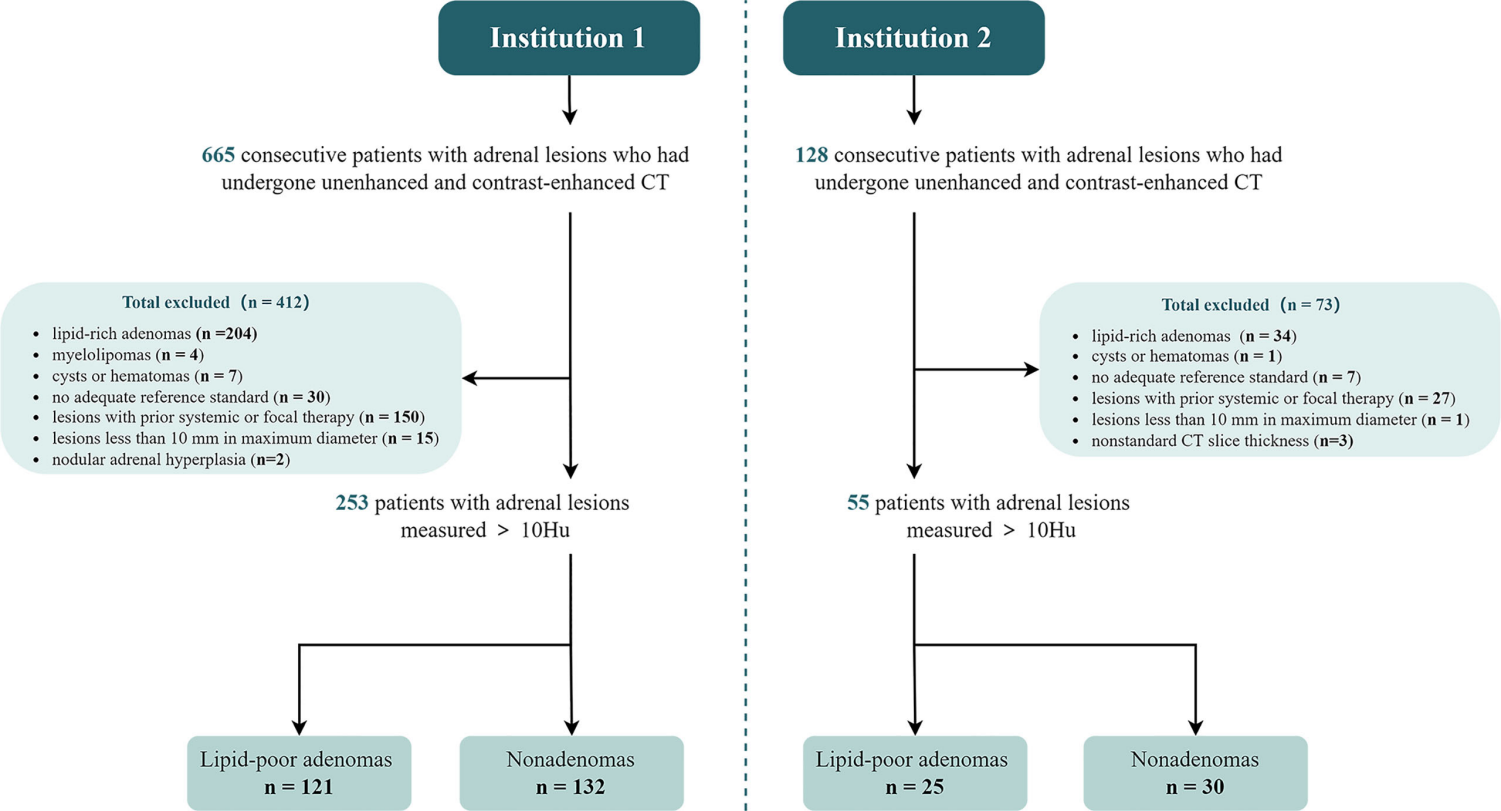
Flowchart of the study sample.

The exclusion criteria were as follows: (a) lesions with an HU ≤ 10 on unenhanced CT and visible lipid-rich lesions (lipid-rich adrenal adenoma or myelolipoma); (b) missing solid components in the lesion: the change of CT attenuation between the portal venous phase and unenhanced phase is ≤ 10 HU; (c) lesions showing an increase of 10%–30% in the maximum diameter of the adrenal gland during the follow-up period; (d) the scheme of the adrenal or abdominal CT did not meet the standards; (e) lesions with a history of systematic or local treatment; and (f) lesions with a maximum diameter < 10 mm, which was determined to avoid the partial volume effect caused by a thickness of 5 mm (8). For patients with multiple adrenal lesions, only the maximum diameter was included in the analysis to reduce the aggregation effect. The flowchart of the patient selection is summarized in **Figure 1**. Patients from Institution 1 were randomly split into the training set and the internal validation set according to a ratio of 7:3. Some data in this study had been used in prior research (16) on radiomics conducted by our team.

### Reference Standard

For all lesions, the final diagnosis was based on pathology or widely accepted imaging standards (8). The diagnostic criteria for lipid-poor adrenal adenomas and nonadenomas are summarized in **Supplementary Material S1**.

### The External Validation Set

Institution 2 analyzed patients with adrenal lesions continuously treated from January 2015 to August 2021, constructed an independent external validation set, and tested the model. Patients in Institution 1 and Institution 2 were selected based on the same inclusion and exclusion criteria (**Figure 1**). Moreover, the scanning equipment, protocol, and contrast agent concentration were consistent for patients in both institutions.

### Image Acquisition

All unenhanced and contrast-enhanced CT images were obtained using multi-slice spiral CT scanners (uCT 530; United Imaging, Shanghai, China; Discovery CT750HD; GE Healthcare, Chicago, IL, USA). The images were displayed and stored in the image archiving and communication system (PACS). The CT protocols are provided in **Supplementary Material S2**.

### Image Analysis

The region of interest was manually drawn in the lesion layer with the maximum diameter. Additionally, the obvious cystic, calcified, and necrotic areas were avoided. All regions of interest were determined on contrast-enhanced images. Subsequently, they were copied to the unenhanced images. Placements were corrected if necessary.

Two radiologists (ZHQ, reader 1, a radiologist with 3 years of experience; LX, reader 2, a radiologist with 20 years of experience), who were blinded to the clinical data and final diagnosis, independently reviewed the CT images and recorded information regarding shape, boundary, and necrosis. Moreover, they measured the maximum diameter and the unenhanced and portal venous phase CT attenuations which was also called contrast-enhanced attenuation.

Absolute enhancement was calculated by subtracting unenhanced from contrast-enhanced attenuation. The absolute enhancement rate was calculated as follows: contrast-enhanced attenuation/unenhanced attenuation × 100%. Based on established guidelines (8): 1 ~ 2 cm, 2 ~ 4 cm, and ≥ 4 cm were defined as small, medium, and large nodules, respectively.

To assess inter-reader agreement, all analyses were performed independently by a third radiologist (PWT, a radiologist with 2 years of experience), who was also unaware of any clinical data and final diagnosis. The consistency of quantitative variables between the two readers was compared using intraclass correlation coefficients (ICC). Inter-reader agreement was evaluated using the kappa statistics for qualitative variables. Between-reader differences were shown in the Bland Altman plots based on the mean of the measurement (17). After the consistency test, the variables with ICC or kappa statistics > 0.8 was included. The average values of the quantitative variables were used for subsequent analysis. Between-reader disagreements in qualitative data were resolved through a consensus between the two readers.

### Statistical Analysis

Normally distributed continuous variables were analyzed using Student’s t-test and presented as means and standard deviations. Non-normally distributed continuous variables were examined using the Mann-Whitney U test and presented as medians and interquartile range (IQR). Categorical variables were analyzed using the chi-square or Fisher’s exact test and expressed as the frequency and percentage.

We conducted a least absolute shrinkage and selection operator (LASSO) algorithm to select demographic variables and CT features in the training set. We established a generalized linear model (logistic regression) through multivariate analysis of statistically significant variables to predict the probability of lipid-poor adrenal adenoma. Identification and calibration are crucial attributes with respect to the performance evaluation of multivariable models (18). Clinical effectiveness was evaluated using decision curve analysis. The constructed model was used to predict the probability of lipid-poor adenoma in the internal validation set and external validation set.

The sensitivity, specificity, accuracy, and receiver operating characteristic curve (AUC) of the model, and two readers were calculated. AUCs between the combined model, unenhanced attenuation, and absolute enhancement rate was compared using the Delong test in the training set and internal validation set. The accuracy between the model and both readers were compared through the McNemar test in the external validation set. MedCalc (version 19.4.1, MedCalc Software) and R software (version 4.1.1, http://www.r-project.org) (rms, glmnet, rmda, ggDCA, Hmisc, DynNom, rsconnect) were used to perform the statistical analyses. Statistical significance was set at two sided. P<0.05.

## Results

### Study Participants

We included 665 patients with adrenal lesions who underwent adrenal or abdominal unenhanced and contrast-enhanced CT. We excluded 412 patients and included 253 in Institution 1 (median age, 55 years [IQR, 47-64 years]; 135 men). Among the included patients, 121 (48%) showed lipid-poor adrenal adenomas, while 132 (52%) showed nonadenomas, including 68 (27%) metastases and 64 (25%) other nonadenomas (**Figure 1**).

The 121 lipid-poor adrenal adenomas were identified based on pathological diagnosis (n = 109); size stability (n = 11), and abnormal ^18^F-FDG uptake, fulfilling the criteria for adenoma (n = 1).

The primary lesions and the diagnostic approaches of metastases, and pathological types of other nonadenomas are presented in **Supplementary Material S3**.

Using the same inclusion and exclusion criteria, 55 patients (median age, 61 years [IQR, 50-70 years]; 29 men) with adrenal lesions from Institution 2 were included in the external validation set. Among them, 25 (45%) had adrenal adenomas and 30 (55%) had nonadenomas, including 13 metastases and 17 others (**Figure 1**).

### Characteristics of the Patients From Institution 1 and Institution 2

**Table 1** summarizes the clinical and CT characteristics of the patients from Institution 1 and Institution 2. Patients with adrenal adenomas were significantly younger and more of the female sex than those with nonadenomas (median age, 52 years [IQR, 44–57 years] vs. 60 years [IQR, 52–67 years], [P < 0.001]; women: 64% [77/121] vs. 31% [41/132], [P < 0.001]). Moreover, patients with adrenal adenomas showed a higher BMI than patients with nonadenomas (median BMI, 24.6 kg/m^2^ [IQR, 22.4–26.7 kg/m^2^] vs. 23.1 kg/m^2^ [IQR, 20.8–25.9 kg/m^2^], [P < 0.001]).

**Table 1:** Characteristics of Institution 1 and Institution 2.

	Institution 1			Comparison with Institution 2		
Variables	Lipid-poor Adenoma (n = 121)	Nonadenoma* (n = 132)	*P*	Institution 1 (n = 253)	Institution 2 (n = 55)	*P*
Age (years), median (IQR)	52 (44-57)	60 (52-67)	**<0.001**	55 (47-64)	61 (50-70)	0.048
Sex, n (%)			**<0.001**			0.932
Male	44 (36)	91 (69)		135 (53)	29 (47)	
Female	77 (64)	41 (31)		118 (47)	26 (53)	
BMI (kg/m^2^), median (IQR)	24.6 (22.4-26.7)	23.1 (20.8-25.9)	**<0.001**	23.5 (21.5-26.2)	23.4 (20.85-25.8)	0.259
Distribution of lesions, n (%)			**0.026**			0.550
Unilateral	102 (84)	96 (73)		198 (78)	41 (75)	
Bilateral	19 (16)	36 (27)		55 (22)	14 (25)	
Necrosis, n (%)	9 (7)	63 (48)	**<0.001**	72 (28)	18 (33)	0.529
Diameter (cm), n (%)			**<0.001**			0.443
1-2	63 (52)	25 (19)		88 (35)	15 (27)	
2-4	54 (45)	62 (47)		116 (46)	29 (53)	
≥4	4 (3)	45 (34)		49 (19)	11 (20)	
Unenhanced attenuation (HU), median (IQR)	23 (16-32)	37 (34-43)	**<0.001**	34 (22-40)	33 (25-37)	0.772
Contrast-enhanced attenuation (HU), median (IQR)	65 (54-76)	67 (57-79)	0.317	66 (57-78)	64 (55-80)	0.691
Absolute enhancement (HU), median (IQR)	35 (25-52)	26 (19-42)	**<0.001**	35 (26-46)	34 (23-50)	0.709
Absolute enhancement ratio (%), median (IQR)	296 (233-353)	175 (157-197)	**<0.001**	214 (173-300)	199 (170-277)	0.679

*There were 132 patients with nonadenoma in the Institution 1, the complete information of them is summarized in **Supplementary Material S3**.

BMI, Body Mass Index; IQR, Interquartile range; kg, kilogram; m, meter. P: categorical variables—Chi-Squared Test or Fisher’s exact test; continuous variables—Mann–Whitney U test. The bold value means statistical significance.

Regarding the CT signs, patients with lipid-poor adrenal adenomas showed lower unenhanced attenuation than patients with nonadenomas (median, 23 HU [IQR, 16–32 HU] vs. 37 HU [IQR, 34–43 HU], [P < 0.001]), with no significant between-group difference in contrast-enhanced attenuation (median, 65 HU [IQR, 54–76 HU] vs. 67 HU [IQR, 57–79 HU], [P = 0.317]). However, patients with adrenal adenomas showed higher absolute enhancement attenuation than patients with nonadenomas (median, 35 HU [IQR, 25–52 HU] vs. 26 HU [IQR, 19–42 HU], [P < 0.001]). Similarly, the absolute enhancement rate was higher in adenomas than in nonadenomas (296% [IQR, 233–353%] vs. 175% [IQR, 157–197%], [P < 0.001]).

Unilateral lesions were more frequent in patients with adenomas than in those with nonadenomas (102 of 121 patients [84%] vs. 96 of 132 patients [73%]; P = 0.026). Lipid-poor adrenal adenomas were smaller in diameter and were less prone to necrosis than nonadenomas (P < 0.001). There were no significant between-group differences in the other demographic and CT characteristics (P > 0.05).

### Characteristics of the Training Set and Internal Validation Set

**Supplementary Table S2** summarizes the clinical and CT characteristics of the training set and internal validation set. Except for the distribution of lesions and contrast-enhanced attenuation, other clinical and CT features were statistically different between patients with adenoma and those with nonadenoma in the training set (P < 0.05). No statistically significant difference was observed in all variables between the training set and the internal validation set.

### Feature Selection

The inter-reader agreement was moderate for shape and boundary (κ = 0.53–0.56) and almost perfect for necrosis (κ = 0.86), size, unenhanced attenuation, and contrast-enhanced attenuation (ICC = 0.98–0.99). More details are shown in **Supplementary Figure S1**. After LASSO, except for body mass index (BMI) and distribution of lesions, the other variables were included (**Supplementary Figure S2**).

### Multivariable Analysis and Model Construction

Multivariate analysis showed that age (odds ratio [OR], 0.94; 95% confidence interval [CI]: 0.90, 0.98; P = 0.015), male sex (OR, 0.26; 95% CI: 0.08, 0.74; P = 0.015), lesion size (2-4cm: OR, 0.51; 95% CI: 0.14, 1.75; P = 0.289; ≥ 4 cm: OR, 0.09; 95% CI: 0.01, 0.57; P = 0.014), necrosis (OR, 0.19; 95% CI: 0.04, 0.78; P = 0.027), unenhanced attenuation (OR, 0.79; 95% CI: 0.72, 0.85; P < 0.001), and contrast-enhanced attenuation (OR, 1.07; 95% CI: 1.04, 1.11; P < 0.001) were independently associated with adrenal adenomas (**Table 2**). The formula of the combined model is as follows:


$$\begin{array}{c} \text{In}\left(\frac{p}{1-p}\right)=7.4743-1.3572\times \left(Sex=male\right)-0.0593\times age-0.6636\times \\ \left(Tumor\;size=Middle\right)-2.4000\times \left(Tumor\;size=Lager\right)-1.6424\\ \left(Necrosis=Yes\right)-0.2361\times Unenhanced\;attenuation\;+0.0700\\ Contrast\;enhanced\;attenuation \end{array}$$

**Table 2:** Results of multivariate analysis for features selected by LASSO algorithm.

Variable	Multivariable Analysis	
	OR (95% CI)	P
Age (per 1 year)	0.94 (0.90, 0.98)	0.015
Sex		
Female	Ref.	
Male	0.26 (0.08, 0.74)	0.015
Diameter (cm)		
1-2	Ref.	
2-4	0.51 (0.14, 1.75)	0.289
≥4	0.09 (0.01, 0.57)	0.014
Necrosis		
No	Ref.	
Yes	0.19 (0.04, 0.78)	0.027
Unenhanced attenuation (per 1 HU)	0.79 (0.72, 0.85)	<0.001
Contrast-enhanced attenuation (per 1 HU)	1.07 (1.04, 1.11)	<0.001

OR, odds ratio; CI, confidence interval; cm, centimeter; HU, Hounsfield Unit; Ref., reference. Data in parentheses are 95% CIs.

### Prognostic Performance of the Model

**Figure 2:**
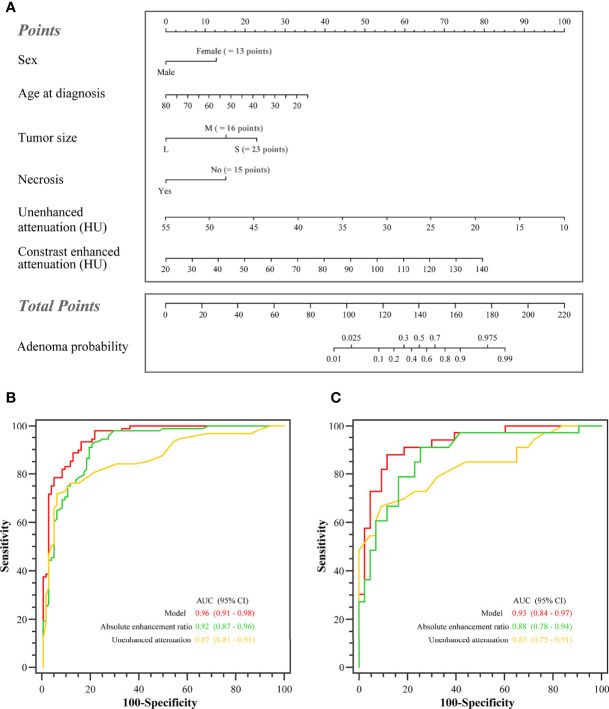
The nomogram and the receiver operating characteristic (ROC) curves in the training set and the internal validation set. **(A)** Nomogram of lipid-poor adrenal adenoma prediction based on clinical-image model. Added up the scores of each variable to get the total score. Based on it, the probability of lipid-poor adrenal adenoma was showed by projecting the score to the risk axis. Online tool is available at https://zhanghuangqi.shinyapps.io/dynnomapp/. **(B)** The ROC curves for differentiating lipid-poor adenomas and nonadenomas in the training set. The highest area under the curve was obtained with the combined model (0.96 [95% CI: 0.91, 0.98]), followed by absolute enhancement rate (0.92 [95% CI: 0.87, 0.96]), and unenhanced attenuation (0.87 [95% CI: 0.81, 0.91]). **(C)** The combined model displayed the best diagnostic performance for prediction of lipid-poor adenomas in the internal validation set (AUC, 0.93 [95% CI: 0.84, 0.97]).

**Figure 3:**
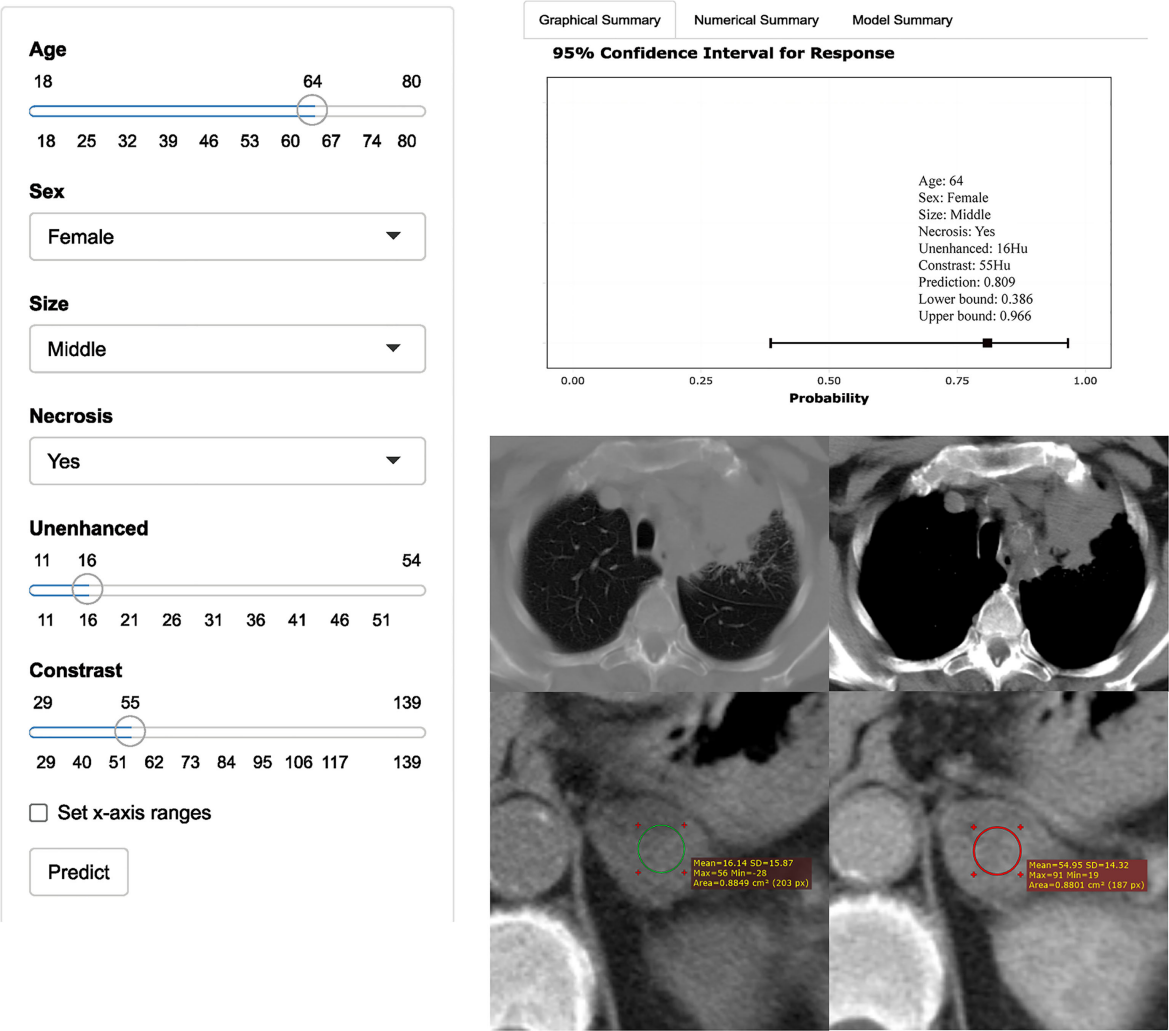
Axial unenhanced and contrast-enhanced adrenal CT images in a 64-year-old woman with cough and expectoration. The woman was accidentally found having left adrenal lesions due to chest CT findings of left upper lung mass and multiple lymph nodes in the left hilar and mediastinum. CT features were analyzed as follows: lesion location = “left”, size = “ middle (2.7cm×2.1cm)”, shape =“quasi-circular”, unenhanced attenuation = 16 HU, contrast-enhanced attenuation = 55 HU, and necrosis= “yes”. Both radiologists evaluated that the possibility of nonadenoma (metastasis) was high, while the possibility of adenoma judged by the model was up to 81% (95% CI: 39%, 97%). The result of pathological diagnosis was adrenal adenoma.

**Figure 4:**
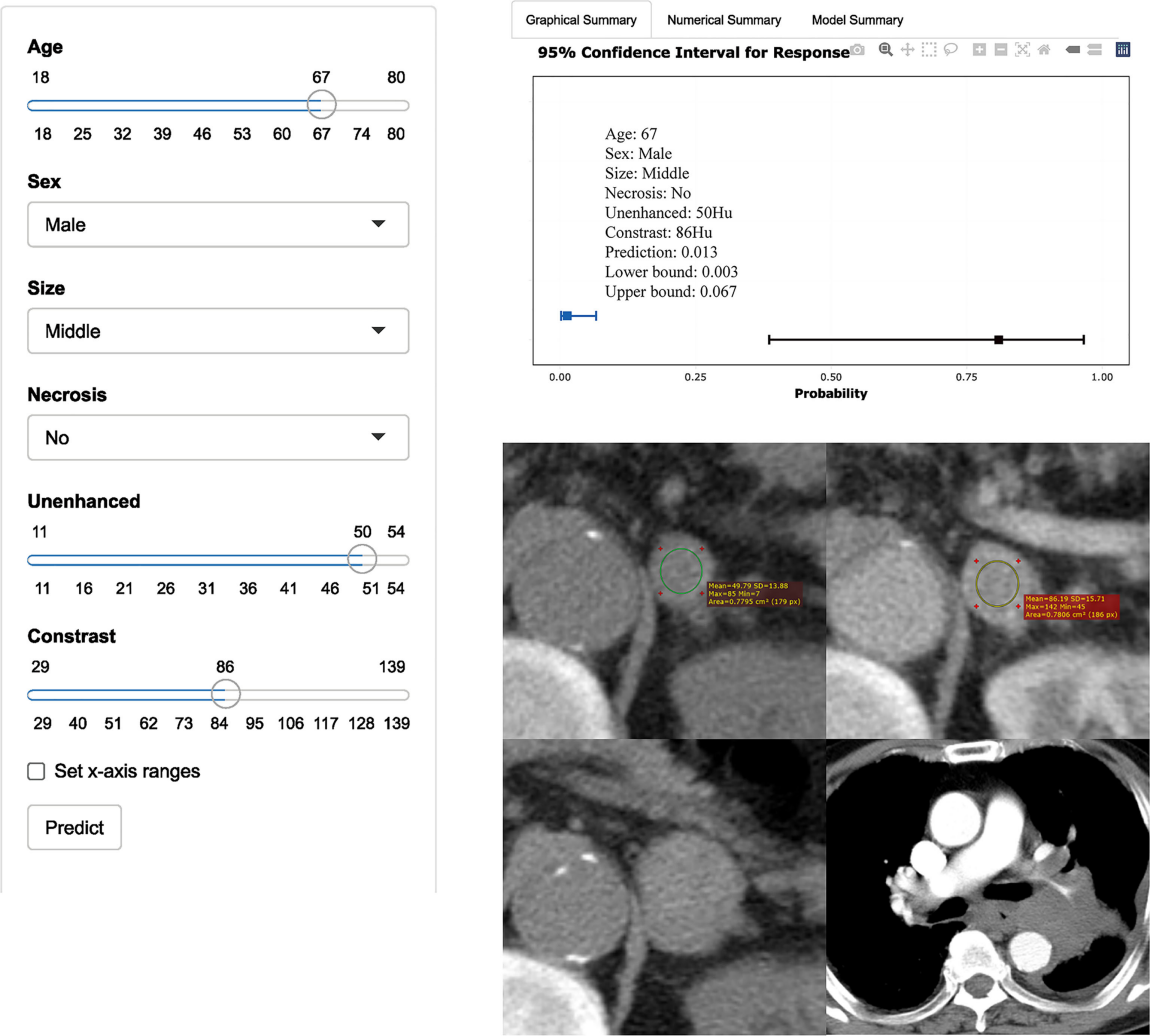
Axial unenhanced and contrast-enhanced adrenal CT images in a 67-year-old man with dizziness and unstable walking. The man was accidentally found having left adrenal lesions due to abdominal CT findings of right kidney occupied. Meanwhile, the boundary of the mass was clear, and no obvious enlarged lymph nodes were found around. CT features were analyzed as follows: lesion location = “left”, size = “middle (2.7cm×2.1cm)”, shape =“quasi-circular”, unenhanced attenuation = 50 HU, and contrast-enhanced attenuation = 86 HU. The renal lesion was considered to be a malignancy. Reader 1 thought that it was more likely to be nonadenoma, while reader 2 thought that it was more likely to be adenoma. The possibility of adenoma judged by the model was only 1% (95% CI: 0%, 7%). One year later, the patient went to see a doctor again due to repeated cough, expectoration, and chest tightness. CT showed that left hilar was occupied by mass with bronchial and pulmonary artery stenosis. It was confirmed as small cell lung cancer pathologically. The left adrenal gland was significantly larger than before, with maximum diameter of 3.3cm. Metastasis was considered first, but it cannot be determined whether the metastasis was from lung cancer or renal cancer.

In the training set, the AUCs were 0.96 (95% CI: 0.91, 0.98), 0.87 (95% CI: 0.81, 0.91), and 0.92 (95% CI: 0.87, 0.96) for the model, unenhanced attenuation, and absolute enhancement rate, respectively. Additionally, the diagnostic performance of the model was higher than that of the unenhanced attenuation or absolute enhancement rate (P < 0.001 and P = 0.002, respectively). The AUC of the model was 0.93 (95% CI: 0.84, 0.97) in the internal validation set, which was superior to that of the unenhanced attenuation and absolute enhancement rate (AUC: 0.83 [95% CI: 0.73, 0.91; P = 0.040] and 0.88 [95% CI: 0.78, 0.94; P = 0.060], respectively). **Figure 2** shows the nomogram and the receiver operating characteristic (ROC) curves in the training set and internal validation set. Moreover, the calibration curve of the model in the training set is shown in **Supplementary Figure S3**. The online tool is available at https://zhanghuangqi.shinyapps.io/dynnomapp/. Examples of the nomogram’s clinical use are displayed in **Figures 3**, **4**. The decision curve and clinical impact curves are shown in **Supplementary Figures S4, S5**. This study revealed that the model achieved a seemingly better net benefit than unenhanced attenuation or relative enhancement rate.

### External Validation Set

In the external validation set (n = 55), the AUC of the model was 0.86 (95% CI: 0.74, 0.94). The model (84%; 95% CI: 71%, 92%) showed a significantly and non-significantly higher accuracy than reader 1 (65%; 95%CI: 51%, 78%; P = 0.031) and reader 2 (69%; 95% CI: 55%, 81%; P = 0.057), respectively (**Table 3**).

**Table 3:** Sensitivity, Specificity, and Accuracy for Differentiating Lipid-Poor Adenomas from Nonadenomas by Two Readers and the Model.

	External Validation Set		
	Sensitivity (%)	Specificity (%)	Accuracy (%)
Reader1	80 (20/25)	53 (16/30)	65 (36/55)
	[59, 93]	[34, 72]	[51, 78]
Reader2	56 (14/25)	80 (24/30)	69 (38/55)
	[35, 76]	[61, 82]	[55, 81]
Model	84 (21/25)	83 (25/30)	84 (46/55)
	[64, 96]	[65, 94]	[71, 92]

Data in parentheses are numbers of lesions, with 95% CIs in brackets.

## Discussion

It is desirable to develop a practical and convenient method for identifying lipid-poor adrenal lesions. To our knowledge, this is the first study to establish a combined model based on easily available demographic information and CT characteristics for differentiating lipid-poor adrenal adenomas from nonadenomas. Our model was validated and tested using the internal validation set and independent external data and showed good diagnostic efficiency. Further, it displayed better diagnostic ability than inexperienced radiologists in the external validation set. To facilitate the clinical use of this model, we have transformed it into online software for use.

Previous studies have tried to identify lipid-poor adrenal adenoma. Several studies found that CT attenuation displayed potential usefulness in distinguishing adrenal adenoma (13, 14, 19). Our clinical-image model showed higher diagnostic efficiency than simple unenhanced CT attenuation and absolute enhancement rates. In previous studies, the diagnosis of adenomas was confirmed by long-term radiological follow-up; there was a relatively low proportion of adenomas confirmed by surgery or puncture (20, 21). However, in our study, 90% (109 of 121 patients) of the adenomas were confirmed by pathology. Yi et al. developed radiomic nomograms to distinguish subclinical pheochromocytoma from lipid-poor adenoma through CT images with an AUC of 0.904 (22). Our model was based on easily available demographic information and CT features and also achieved good performance; moreover, our study covered a wider etiology of adrenal nonadenoma.

Multivariable analysis revealed that the main demographics for predicting lipid-poor adrenal adenoma were age and sex, consistent with previous studies (23–25). Many nonadenomas were metastases, which tend to occur in the elderly (8). The presence or absence of necrosis and lesion size were independently associated with the diagnosis of lipid-poor adrenal adenoma. According to relevant guidelines (8), we used the lesion size as a categorical variable since it is clinically significant. Given the high possibility of benign lesions, follow-up should be conducted for lesions with a size of 1–2 cm. For lesions with a size > 2 cm and < 4 cm, the next plan is determined in combination with unenhanced attenuation. Finally, for lesions larger than 4 cm, surgical resection is decided based on the malignancy history.

Patients with adenomas showed lower unenhanced attenuation than patients with nonadenomas. Several studies have demonstrated that adrenal adenomas have lower unenhanced attenuation than pheochromocytomas or malignant adrenal lesions (26). Pathologically, adrenal adenoma, whether rich in or lacking lipids, is a benign neoplasm of adrenocortical cells. The adrenal cortex consists of zona glomerulosa, zona fasciculata, and zona reticularis. The zona fasciculata constitutes three-fourths of the cortex comprising lipid-laden cells (1).

Although adenomas have been reported to show rapid wash in the portal vein phase (13, 27), they often showed no statistically significant difference in univariate analysis. A recent study (12) indicated that the ratio of portal venous phase attenuation to unenhanced attenuation allowed sufficient identification of lipid-poor adenomas and nonadenomas. Therefore, we attempted to incorporate the portal vein phase attenuation into the combined model, which was an independent risk factor in multivariate analysis. The above may be attributed to the correlations between independent variables; the influence of independent variables on dependent variables reflects their own role and the mixed roles of other variables.

In the external validation set, there were 13 cases of adrenal metastases, which were accurately identified by our combined model. A total of five nonadenomas were misjudged as adenomas, including four pheochromocytomas and one spindle cell tumor. Only one pheochromocytoma among these five patients was correctly identified by reader 2. Previous studies have indicated that some pheochromocytomas are misdiagnosed as adenomas on adrenal enhanced CT (20, 28). Furthermore, these pheochromocytomas were rich in blood vessels and displayed rapid washout similar to adenomas, which cannot be accurately characterized in the delayed phase (20, 28).

Our study has some limitations. First, we did not collect the full clinical history or laboratory examination. However, this was consistent with our study objective, which was an early, rapid, and noninvasive diagnosis of lipid-poor adenoma and effective stratification of patients to avoid some unnecessary examination. Second, we used a total iodine dose of 400 mgI/kg, which is lower than previously reported values (10, 20, 29) and might limit the application of our model. This could be attributed to our participants weighing less than those in previous studies due to race differences. Simultaneously, an excessively high iodine dose adversely affects patients, causing fever, pain, and contrast medium nephropathy (30, 31). Third, the diagnostic efficiency of the combined model in the external validation set was not as high as we expected. Moreover, the diagnostic accuracy of the two readers in the external validation set was lower than that in the training set, which could partly explain the reduced diagnostic efficiency of the model. Specifically, this might be attributed to differences in the types of patients treated in both sets.

## Conclusion

This study shows that the combined model, which is based on accessible demographic characteristics and CT features, can facilitate the identification of lipid-poor adrenal adenoma. In the training set, the combined model had better diagnostic efficiency than unenhanced attenuation or the absolute enhancement rate. In the external validation set, the model showed higher accuracy than an inexperienced radiologist.

## Data Availability Statement

The original contributions presented in the study are included in the article/**Supplementary Material**. Further inquiries can be directed to the corresponding authors.

## Author Contributions

WP, HZ, and WJ: conception and design. HZ, XL, SJ, and JY: collection and assembly of data. HZ, WP, and LM: data analysis and interpretation. WP, XD, BZ, and JY: manuscript writing. All authors contributed to the article and approved the submitted version.

## Conflict of Interest

Author LM is employed by He Kang Corporate Management (SH).

The remaining authors declare that the research was conducted in the absence of any commercial or financial relationships that could be construed as a potential conflict of interest.

## Publisher’s Note

All claims expressed in this article are solely those of the authors and do not necessarily represent those of their affiliated organizations, or those of the publisher, the editors and the reviewers. Any product that may be evaluated in this article, or claim that may be made by its manufacturer, is not guaranteed or endorsed by the publisher.

## Abbreviations

CT, computed tomography; AUC, area under the receiver operating characteristic curve; CI, confidence interval; IQR, Interquartile range; BMI, Body Mass Index; HU, Hounsfield unit.
