# Supplementary material for: Development and Validation of a Clinical-Image Model for Quantitatively Distinguishing Uncertain Lipid-Poor Adrenal Adenomas From Nonadenomas
Source: Front Oncol. 2022 Jul 13;12:902991. doi: 10.3389/fonc.2022.902991 (PMC9326106; doi:10.3389/fonc.2022.902991)
Supplement: Supplementary file 1 [file DataSheet_1.pdf]

Supplementary Material 1

Those who met the following criteria were diagnosed as lipid-poor adrenal adenoma: (1a) pathologic diagnosis; (1b) size stability within 12 months of imaging follow-up (transverse diameter increases less than 10%).

Patients with extraadrenal malignancies who met the following criteria were diagnosed as metastases: (2a) pathological diagnosis; (2b) newly discovered or increased volume (maximum diameter increased by at least 30%) within 12 months of follow-up or decreased volume within the interval after systemic chemotherapy, or (2c) abnormal high uptake of ^{18}F-FDG.

Other circumstances: (3a) all nonadenomas except metastases were confirmed by pathology, such as pheochromocytoma, adrenocortical carcinoma and so on; (3b) the lesion showed abnormal ^{18}F-FDG uptake, but met the criteria of adenoma was diagnosed as adenoma.
