# Supplementary material for: Development and Validation of a Clinical-Image Model for Quantitatively Distinguishing Uncertain Lipid-Poor Adrenal Adenomas From Nonadenomas
Source: Front Oncol. 2022 Jul 13;12:902991. doi: 10.3389/fonc.2022.902991 (PMC9326106; doi:10.3389/fonc.2022.902991)
Supplement: Supplementary file 2 [file DataSheet_2.pdf]

Supplementary Material 2

The CT protocols of Discovery CT750 HD were as follows: collimation，64×0.625mm；rotation time，0.6 seconds; pitch factor, 0.984; 120 kVp, 180–300 mAs with automatic tube current modulation. The CT protocols of uCT 530 were as follows: collimation, 40×0.55mm；rotation time, 0.8 s; pitch factor, 1.075; 120 kVp, 180–300 mAs with automatic tube current modulation. Each patient received 300/350mgI/mL of contrast material (iohexol or iodopanol) inserted into an anterior elbow veins and the total iodine dose was 400mgI/ kg. By using the automatic injection tracking technology with automatic scanning trigger software, the portal venous phase image was obtained 45 seconds after the level of abdominal aorta reached the trigger threshold (100HU). The image was reconstructed to 2.5-mm or 5-mm thickness by standard algorithm, and then uploaded to the image archiving and communication system (PACS).
