# Supplementary material for: Development and Validation of a Clinical-Image Model for Quantitatively Distinguishing Uncertain Lipid-Poor Adrenal Adenomas From Nonadenomas
Source: Front Oncol. 2022 Jul 13;12:902991. doi: 10.3389/fonc.2022.902991 (PMC9326106; doi:10.3389/fonc.2022.902991)
Supplement: Supplementary file 3 [file DataSheet_3.pdf]

Supplementary Material 3

The primary tumors of the adrenal metastases included lung cancer (n = 36), gastric or esophageal cancer (n = 8), colorectal cancer (n = 5), hepatic cancer (n = 5), kidney cancer (n = 3), ovarian cancer (n = 2), gallbladder carcinoma (n = 1), pancreatic cancer (n = 1), melanoma (n = 1), breast cancer (n = 1), cervical cancer (n = 1), and unknown malignancy (n = 4). Other nonadenomas were pheochromocytoma (n=41), adrenocortical carcinoma (n = 7), lymphoma(n=6), ganglioneuroma (n = 6), vascular tumors(n=3) and spindle cell tumor (n = 1).

The diagnostic methods of metastases include pathological diagnosis (n = 8), volume increase newly found or within 12 months of follow-up (n = 27), volume reduction in the interval after systemic chemotherapy (n = 20), and abnormal high ^{18}F-FDG uptake (n = 13). All nonadenomas except metastases were confirmed by pathology.
