# Supplementary material for: Development and Validation of a Clinical-Image Model for Quantitatively Distinguishing Uncertain Lipid-Poor Adrenal Adenomas From Nonadenomas
Source: Front Oncol. 2022 Jul 13;12:902991. doi: 10.3389/fonc.2022.902991 (PMC9326106; doi:10.3389/fonc.2022.902991)
Supplement: Supplementary file 4 [file Image_1.pdf]

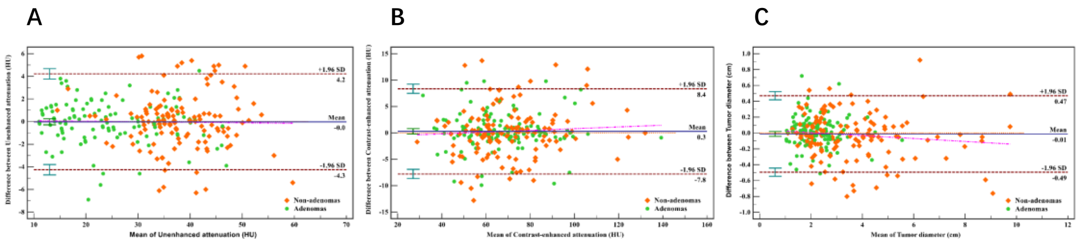

Supplementary Figure 1 | Bland–Altman plots of (A) unenhanced attenuation, (B) contrast-enhanced attenuation and (C) the diameter of the lesions for interrater agreement analysis. Adenomas and nonadenomas are represented by green dots and yellow diamonds, respectively. Bland-Altman plots include the mean difference (as solid blue lines) and 95% limits of agreement (as dashed red lines).

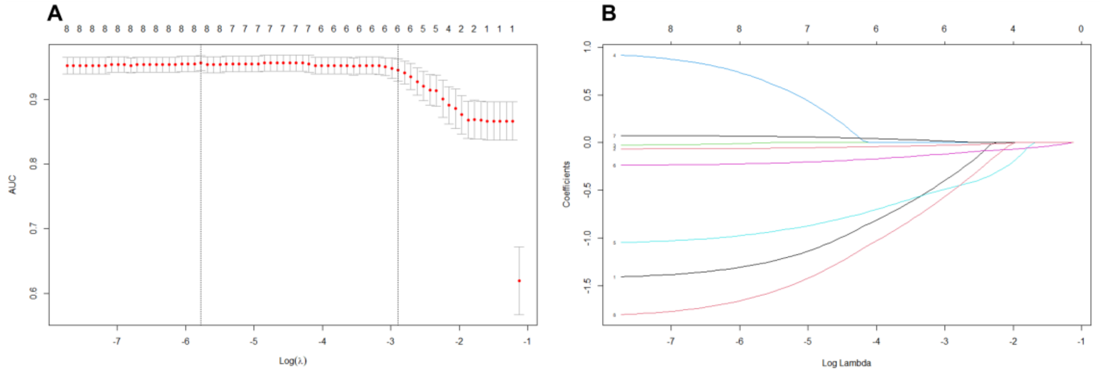

Supplementary Figure 2 | (A) The cross-validation results. The area under the curve (AUC) changed alongside log(λ). Lambda.min was the value of λ that gave minimum error, while lambda.1se was the value of λ that gave the most regularized model such that the error is within one standard error of the minimum and the least number of independent variables. We got the value of lambda.1se (lambda.1se = 0.05535013). The model built by lambda.1se was the simplest and corresponding AUC was relatively high. (B) The estimated coefficients for least absolute shrinkage and selection operator (LASSO) algorithm.

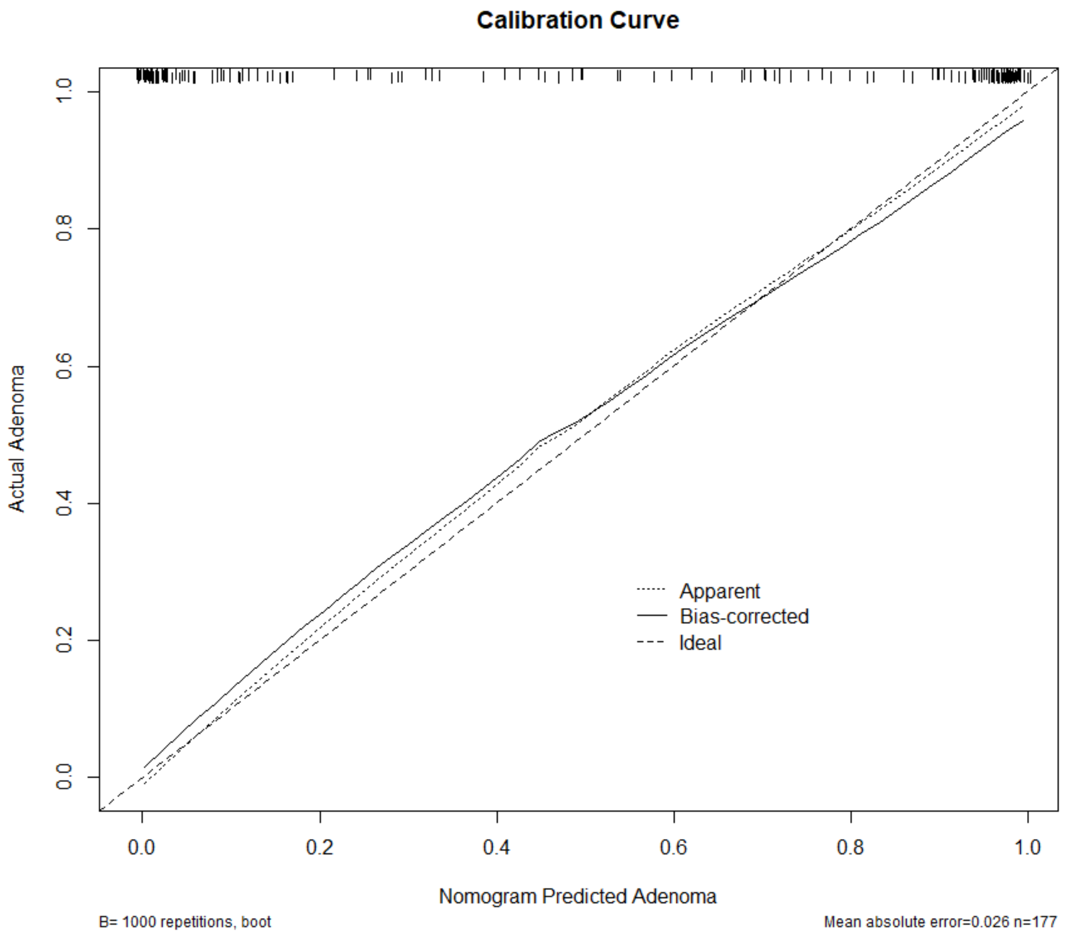

Supplementary Figure 3 | Calibration curve of the model in the training set.

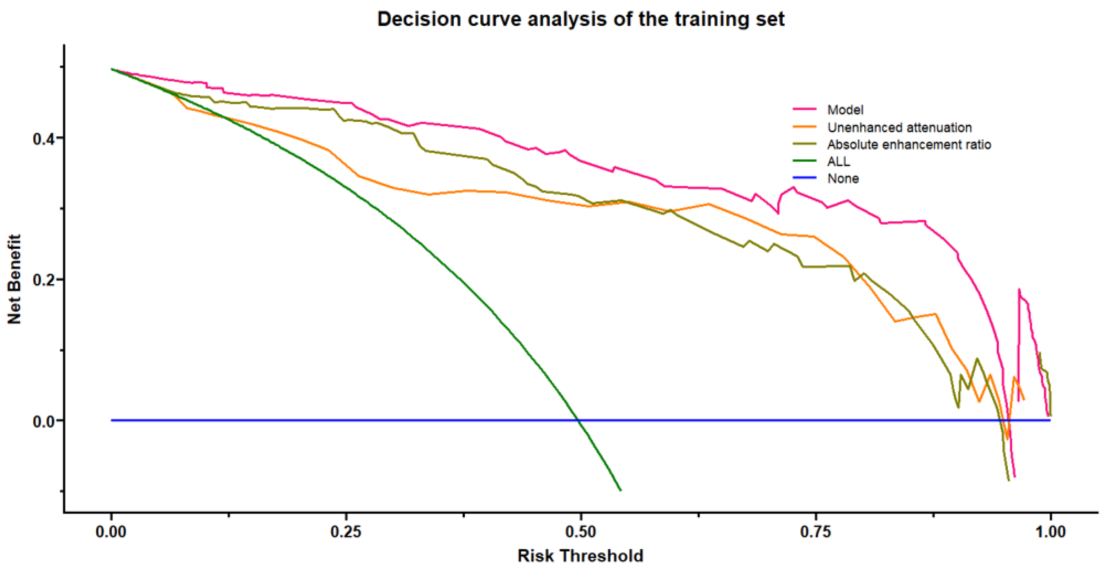

Supplementary Figure 4 | Decision curves of the combined model, absolute enhancement rate, and unenhanced attenuation for differentiating lipid-poor adenomas and nonadenomas in the training set. The decision curve shows the clinical practicability based on the threshold (x-axis) for predicting lipid-poor adrenal adenoma and the net benefit (y-axis) of stratification of patients using the model.

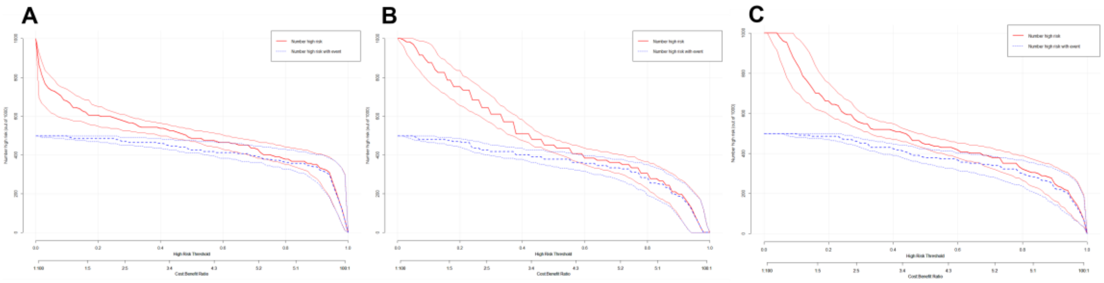

Supplementary Figure 5 | Clinical impact curves of combined model, unenhanced attenuation and absolute enhancement rate, respectively, were plotted in (A, B, and C, respectively). The number of high-risk patients and the number of high-risk patients with the event (lipid-poor adrenal adenoma) were shown at different threshold probabilities.
