# Supplementary material for: Development and Validation of a Clinical-Image Model for Quantitatively Distinguishing Uncertain Lipid-Poor Adrenal Adenomas From Nonadenomas
Source: Front Oncol. 2022 Jul 13;12:902991. doi: 10.3389/fonc.2022.902991 (PMC9326106; doi:10.3389/fonc.2022.902991)
Supplement: Supplementary file 5 [file Table_1.pdf]

TRIPOD Checklist: Prediction Model Development and Validation

Section	Item		Checklist description	Reported on Page Number/Line Number	Reported on Section/Paragraph
**Title and abstract**					
Title	1	D;V	Identify the study as developing and/or validating a multivariable prediction model, the target population, and the outcome to be predicted.	Page 1/Line 1-3.	"Development and Validation of a [illegible]
Abstract	2	D;V	Provide a summary of objectives, study design, setting, participants, sample size, predictors, outcome, statistical analysis, results, and conclusions.	Page 2/Line 21-46.	"Background: There remains a demand for a [illegible]
**Introduction**					
Background and objectives	3a	D;V	Explain the medical context (including whether diagnostic or prognostic) and rationale for developing or validating the multivariable prediction model, including references to existing models.	Page 3/Line 48-67.	"Over the last decades, there has been an epidemic [illegible]
	3b	D;V	Specify the objectives, including whether the study describes the development or validation of the model or both.	Page 3/Line 67-68.	" Therefore, we aimed to develop a practical [illegible]
**Methods**					
Source of data	4a	D;V	Describe the study design or source of data (e.g., randomized trial, cohort, or registry data), separately for the development and validation data sets, ifapplicable.	Page 3-4/Line 74-99.	"We conducted a retrospective study on [illegible]
	4b	D;V	Specify the key study dates, including start of accrual; end of accrual; and, if applicable, end of follow-up.	Page 3/Line 74-75.	"We conducted a
Participants	5a	D;V	Specify key elements of the study setting (e.g., primary care, secondary care, general population) including number and location of centres.	Page 3-4/Line 74-75 and Line 95-96.	" We conducted a retrospective study on [illegible]
	5b	D;V	Describe eligibility criteria for participants.	Page 3-4/Line 75-86.	"The inclusion criteria [illegible]
	5c	D;V	Give details of treatments received, if relevant.	N/A	Not relevant.
Outcome	6a	D;V	Clearly define the outcome that is predicted by the prediction model, including how and when assessed.	Page 4/Line 91-93.	" the final diagnosis was based on pathology or [illegible]
	6b	D;V	Report any actions to blind assessment of the outcome to be predicted.	Page 5/Line 110-114	"Two radiologists (ZHQ,
Predictors	7a	D;V	Clearly define all predictors used in developing or validating the multivariable prediction model, including how and when they were measured.	Page 5/Line 112-118.	"reviewed CT images and recorded information [illegible]
	7b	D;V	Report any actions to blind assessment of predictors for the outcome and other predictors.	Page 5/Line 119-121.	" To assess inter-reader
Sample size	8	D;V	Explain how the study size was arrived at.	Page 3-4/Line 74-75 and	"We conducted a

Missing data	9	D;V	Describe how missing data were handled (e.g., complete-case analysis, single imputation, multiple imputation) with details of any imputation method.	N/A	After data exclusion, the included data were
Statistical analysis methods	10a	D	Describe how predictors were handled in the analyses.	Page 5-6/Line 129-147.	"Normally distributed
	10b	D	Specify type of model, all model-building procedures (including any predictor selection), and method for internal validation.	Page 5-6/Line 134-140.	"We conducted a least absolute shrinkage and
	10c	V	For validation, describe how the predictions were calculated.	Page 6/Line 139-140.	" The constructed model
	10d	D;V	Specify all measures used to assess model performance and, if relevant, to compare multiple models.	Page 6/Line 141-145.	"The sensitivity,
	10e	V	Describe any model updating (e.g., recalibration) arising from the validation, if done.	N/A	Not need model updating
Risk groups	11	D;V	Provide details on how risk groups were created, if done.	N/A	No risk groups in the
Development vs. validation	12	V	For validation, identify any differences from the development data in setting, eligibility criteria, outcome, and predictors.	Page 4/Line 96-98	"Patients in the Institution 1 and Institution 2 were
**Results**					
Participants	13a	D;V	Describe the flow of participants through the study, including the number of participants with and without the outcome and, if applicable, a summary of the follow-up time. A diagram may be helpful.	Figure 1.	Figure 1.
	13b	D;V	Describe the characteristics of the participants (basic demographics, clinical features, available predictors), including the number of participants with missing data for predictors and outcome.	Table 1, Supplementary Table 2 and Page 7-8/Line	"Table 1 summarizes the clinical and CT
	13c	V	For validation, show a comparison with the development data of the distribution of important variables (demographics, predictors and outcome).	Table 1 and Supplementary Table 2.	Table 1 and Supplementary Table 2.
Model development	14a	D	Specify the number of participants and outcome events in each analysis.	Figure 1.	Figure 1.
	14b	D	If done, report the unadjusted association between each candidate predictor and outcome.	Table 1 and	Table 1 and
Model specification	15a	D	Present the full prediction model to allow predictions for individuals (i.e., all regression coefficients, and model intercept or baseline survival at a given time point).	Figure 2.	Figure 2.
	15b	D	Explain how to the use the prediction model.	Figure 2, and Page 9/Line	"Figure 2 shows the
Model performance	16	D;V	Report performance measures (with CIs) for the prediction model.	Page 9/Line 209-215.	Figure 2, and Table 3.
Model-updating	17	V	If done, report the results from any model updating (i.e., model specification, model performance).	N/A	Not need model-updating.
**Discussion**					
Limitations	18	D;V	Discuss any limitations of the study (such as nonrepresentative sample, few events per predictor, missing data).	Page 11/Line 276-287.	"Our study has some limitations. First...."

Interpretation	19a	V	For validation, discuss the results with reference to performance in the development data, and any other validation data.	Page 11/Line 269-275.	"In the external validation set, there were 13 cases of adrenal metastases, which
	19b	D;V	Give an overall interpretation of the results, considering objectives, limitations, and results from similar studies, and other relevant evidence.	Page 9-12/Line 229-287.	"To our knowledge, this is the first study to establish [illegible]
Implications	20	D;V	Discuss the potential clinical use of the model and implications for future research.	Page 12/Line 230-235.	"this is the first study to
**Other information**					
Supplementary information	21	D;V	Provide information about the availability of supplementary resources, such as study protocol, Web calculator, and data sets.	Supplementary Material	Supplementary Material
Funding	22	D;V	Give the source of funding and the role of the funders for the present study.	N/A	No funding.

* Items relevant only to the development of a prediction model are denoted by D, items relating solely to a validation of a prediction model are denoted by V, and items relating to both are denoted D;V. We recommend using the TRIPOD Checklist in conjunction with the TRIPOD Explanation and Elaboration document.
