# Supplementary material for: Development and Validation of a Clinical-Image Model for Quantitatively Distinguishing Uncertain Lipid-Poor Adrenal Adenomas From Nonadenomas
Source: Front Oncol. 2022 Jul 13;12:902991. doi: 10.3389/fonc.2022.902991 (PMC9326106; doi:10.3389/fonc.2022.902991)
Supplement: Supplementary file 6 [file Table_2.pdf]

SUPPLEMENTARY TABLE 2 | Characteristics of the training set and the internal validation set

	Training set			Comparison with internal validation set		
	Lipid-poor	Nonadenoma*		Training set	internal validation set	
**Variables**	Adenoma(n=88)	(n=89)	***P***	(n=177)	(n=76)	***P***
Age (years), median (IQR)	51 (41-56)	60 (53-67)	**< 0.001**	55 (47-64)	61 (50-70)	0.554
Sex, n (%)			**< 0.001**			0.879
Male	35 (40)	60 (67)		95 (54)	40 (53)	
Female	53 (60)	29 (32)		82 (46)	36 (47)	
BMI (kg/m^2), median (IQR)	24.5 (22.2-26.7)	23.1 (20.9-26.0)	**0.023**	23.5 (21.5-26.2)	23.4 (20.9-25.8)	0.401
Distribution of lesions, n (%)			0.051			0.862
Unilateral	74 (84)	64 (72)		138 (78)	60 (79)	
Bilateral	14 (16)	25 (28)		39 (22)	16 (21)	
Necrosis, n (%)	6 (7)	40 (45)	**< 0.001**	46 (26)	26 (34)	0.184
Diameter (cm), n (%)			**< 0.001**			0.839
1-2	47 (53)	16 (18)		63 (35)	25 (33)	
2-4	38 (43)	41 (46)		79 (45)	37 (49)	
≥4	3 (3)	32 (36)		35 (20)	14 (18)	
Unenhanced attenuation (HU), median (IQR)	21 (15-30)	38 (34-43)	**< 0.001**	34 (22-40)	33 (25-37)	0.402
Contrast-enhanced attenuation (HU), median (IQR)	62 (53-76)	66 (57-79)	0.348	66 (57-78)	64 (55-80)	0.273
Absolute enhancement (HU), median (IQR)	40 (35-49)	25 (21-37)	**< 0.001**	35 (26-46)	34 (23-50)	0.732
Absolute enhancement ratio (%), median (IQR)	300 (238-352)	171 (155-196)	**< 0.001**	214 (173-300)	199 (170-277)	0.612

** There were 89 patients with nonadenoma in the training set.*

BMI, Body Mass Index; IQR, Interquartile range; kg, kilogram; m, meter. P: categorical variables—Chi-Squared Test or Fisher's exact test; continuous variables—Mann–Whitney U test. The bold value means statistical significance.
